# Primary Isolated Lymphoma of the Fourth Ventricle in an Immunocompetent Patient

**DOI:** 10.1155/2013/614658

**Published:** 2013-03-28

**Authors:** Rakan Bokhari, Ahmad Ghanem, Mahmoud Alahwal, Saleh Baeesa

**Affiliations:** ^1^Division of Neurological Surgery, Faculty of Medicine, King Abdulaziz University, P.O. Box 80215, Jeddah 21589, Saudi Arabia; ^2^Department of Pathology, Faculty of Medicine, King Abdulaziz University, P.O. Box 80215, Jeddah 21589, Saudi Arabia; ^3^Department of Medicine, Faculty of Medicine, King Abdulaziz University, P.O. Box 80215, Jeddah 21589, Saudi Arabia

## Abstract

Primary central nervous lymphoma (PCNSL) is a rare variant of extranodal non-Hodgkin's lymphoma with a especially poor prognosis. The diagnosis is usually encountered in immunodeficient patients but is also encountered, albeit uncommonly, in the immunocompetent. We present a 50-year-old male who developed signs and symptoms of increased intracranial pressure. Imaging revealed the presence of a fourth ventricle mass with obstructive hydrocephalus. First, the patient underwent emergency endoscopic third ventriculostomy followed, few days later, by complete tumor resection via a posterior fossa craniotomy. Postoperative histopathology revealed the lesion to be a PCNSL. He received adjuvant chemotherapy and radiation and remained with no recurrence on regular imaging studies for 18-month followup. We report herein the fourth case of isolated PCNSL lesion to the fourth ventricle in the literature and provide the rationale for our belief that craniotomy and tumor resection, if feasible, should be the initial line of management in similar cases to relieve hydrocephalus and achieve the diagnosis.

## 1. Introduction

Primary central nervous system lymphoma (PCNSL) is a rare variant of extranodal non-Hodgkin's lymphoma (NHL) with an uncertain origin and pathogenesis [1, 2]. It accounts for about 1% of all primary brain tumors and is most commonly a diffuse large B-cell lymphoma [1, 2]. PCNSL is usually associated with immunodeficiency (most commonly HIV) but is increasingly observed in immunocompetent patients [2]. We present what is, to the authors' best knowledge, the fourth case of PCNSL isolated to the fourth ventricle and the eleventh case of solely intraventricular PCNSL [3–5]. We attempt to raise awareness of this rare entity and describe our experience with this rare entity. Unlike the consensus towards PCNSL in general, we believe that surgical resection, if feasible, in similar cases presenting with hydrocephalus is the optimal line of management [6, 7].

## 2. Case Report

A 50-year-old male physician, with no medical comorbidities, presented to the emergency room with history of forceful unrelenting vomiting associated with moderate nausea that had progressed over the past 2 weeks. Initially attributed to gastritis, his symptoms did not improve with proton pump inhibitor and antiemetic medications. 

His level of consciousness had started to drop over the preceding 24 hours with significant increase in the severity of his headache. Physical exam showed a depressed Glasgow coma score (GCS) of 12 equally reactive pupils with no focal neurological deficit.

**Figure 1:**
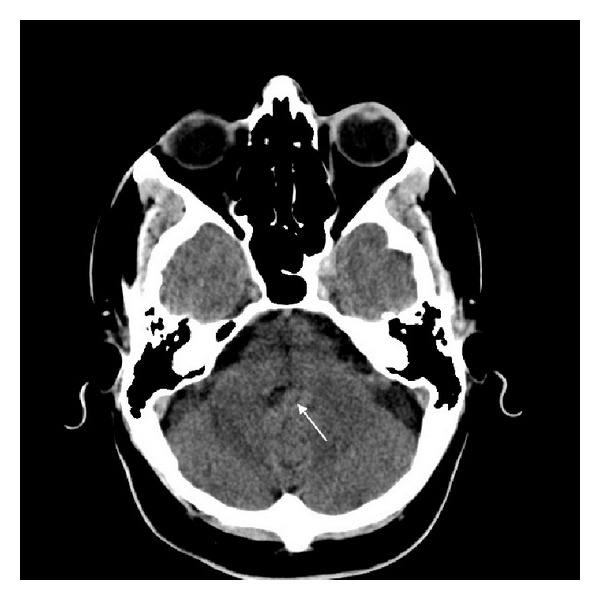
Brain CT scan demonstrating hyperdense tumor (arrow) filling the 4th ventricle.

An urgent brain computed tomography (CT) scan revealed obstructive hydrocephalus with an infratentorial somewhat hyperdense lesion in the fourth ventricular that enhanced homogeneously (Figure 1).

The patient was admitted and urgent endoscopic third ventriculostomy was performed, with rapid recovery of his level of consciousness postoperatively. Cerebrospinal fluid (CSF) analysis and cytology results were within normal and negative for malignant cells.

**Figure 2:**
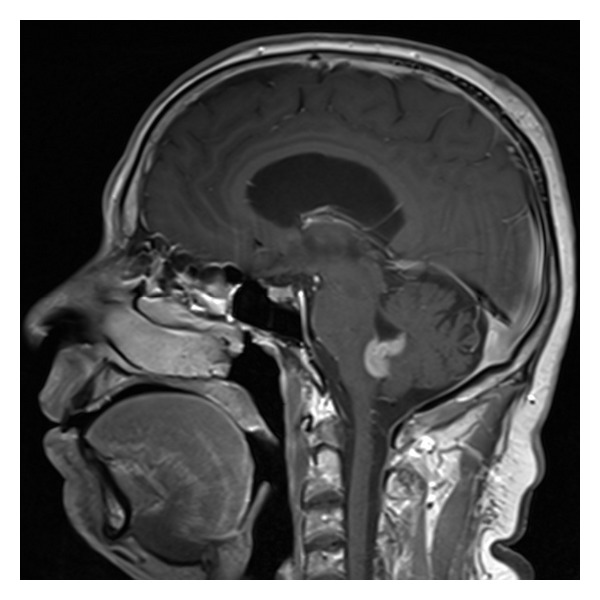
Sagittal T1-WI demonstrating 20 × 25 × 30 mm intensely enhancing fourth ventricular tumor with obstructive hydrocephalus.

**Figure 3:**
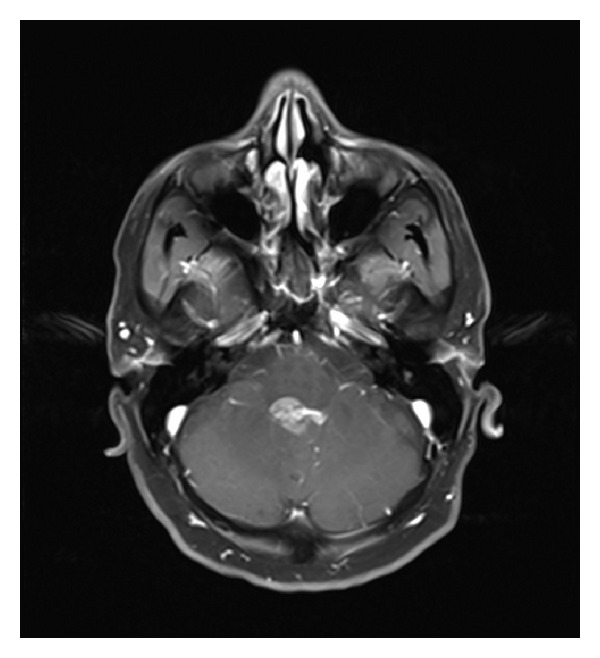
Axial T1-WI demonstrating 20 × 25 × 30 mm intensely enhancing fourth ventricular tumor with obstructive hydrocephalus.

Further brain and spine imaging with magnetic resonance (MR) scan demonstrated a 20 × 25 × 30 mm strongly enhancing mass occupying the inferior half of the fourth ventricle (Figures 2 and 3). The mass acquired the shape of the ventricular cavity, extended towards the lateral foramina of Luschka and inferiorly towards the obex without evidence of fourth ventricular dilation. No evidence of parenchymal invasion or drop metastases was seen on imaging of the whole spine. The radiologist's impression was that of an ependymoma or possibly a subependymoma.

The patient was then shifted, 2 days later, to the operating theatre for resection of a tumor of the fourth ventricle. Midline posterior fossa craniotomy was performed and the tumor was accessed through a transvermian approach. The tumor was exophytic, grayish in color, firm, and fairly vascular, being fed by leptomeningeal vessels arising from top of the brainstem. Invasion of the lower pons and upper medulla oblongata in addition to the superior vermis was noted intraoperatively.

Initially, biopsy was taken and frozen section was suggestive of malignant neoplasm with differential diagnosis including medulloblastoma and ependymoma. Complete resection was proceeded using ultrasonic aspiration and was attainable utilizing an easily identifiable tumor-brainstem interface.

**Figure 4:**
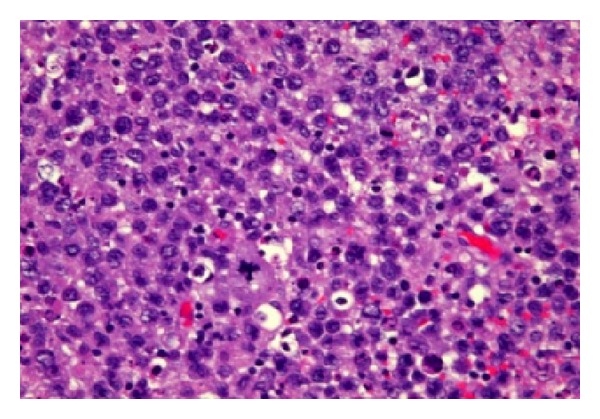
Hematoxylin and Eosin stain showing neoplastic growth formed of highly pleomorphic cells with hyperchromasia, irregular nuclear membranes, vesicular nucleus, and prominent nucleoli. Aggregations of mature lymphocytes also seen, with areas of hemorrhagic necrosis apoptosis and calcification seen.

**Figure 5:**
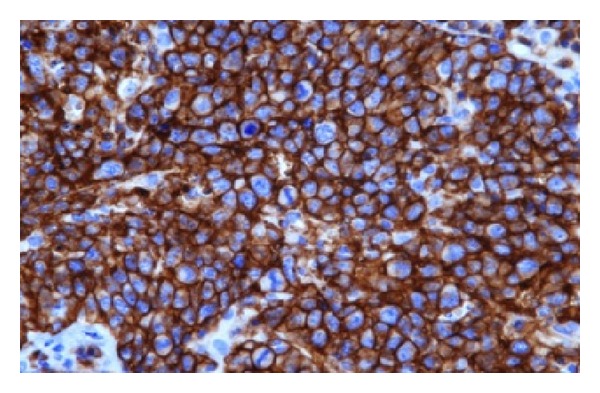
Immunohistochemical stains were positive for CD45 (Leukocyte Common Antigen).

Histopathology would show the cells positive for CD45 (Leukocyte Common Antigen) as well as for CD79a, CD20 (B-cell antigens), and HLA-DR and focally positive for CD10 and vimentin. The morphology and immunohistochemical profile was consistent with a high-grade B-cell lymphoma (Figures 4 and 5). 

Consequent to receiving the histopathology results, staging workup consisting of neck, chest, abdomen, and pelvis CT studies would show no disease elsewhere and HIV tests were negative.

The patient has a smooth postoperative period and his vomiting and gait imbalance had markedly improved.

**Figure 6:**
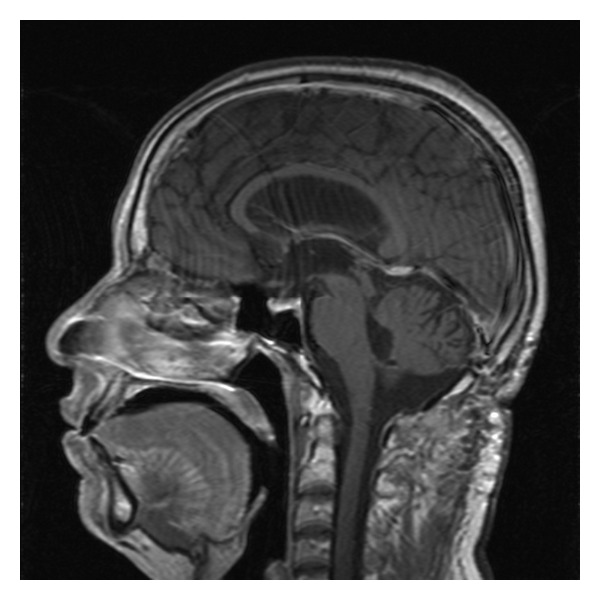
Enhanced sagittal T1-WI demonstrating no residual tumor.

**Figure 7:**
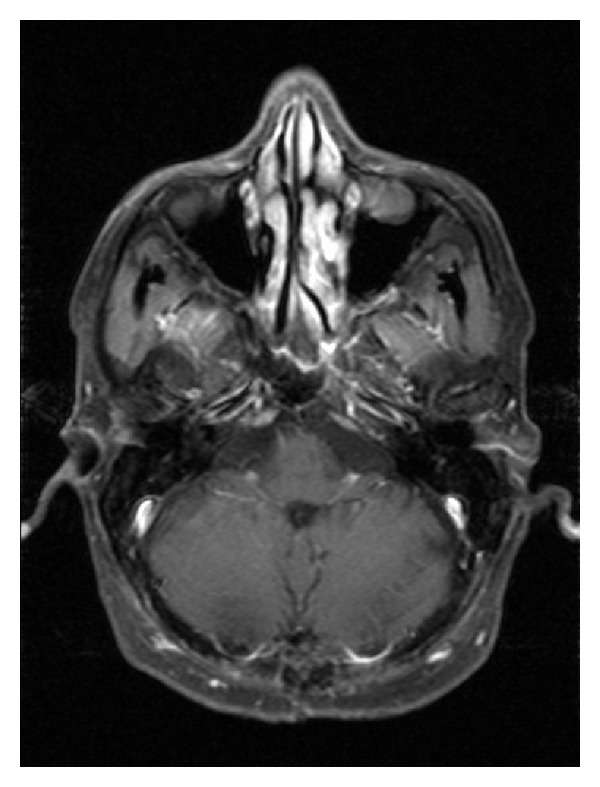
Enhanced axial T1-WI demonstrating no residual tumor.

He received a 6 cycles of intravenous and intrathecal methotrexate with subsequent whole brain radiotherapy and boost to the surgical bed of total of 30 Gys. Both immediate and 18-month postoperative brain and whole spine MR imaging scans showed no recurrence (Figures 6 and 7).

## 3. Discussion

Central nervous system (CNS) lymphomas are considered by neurosurgeons as nonsurgical tumors due to their diffuse infiltration and exquisite sensitivity to chemoradiation [2]. In fact, attempted resection or decompression has been shown to be of no benefit, and possibly harmful [6, 7]. The preoperative identification of these tumors is therefore of great relevance, as their suspicion changes the goal of surgery from a potentially morbid resection to a minimally invasive biopsy for tissue diagnosis and initiation of appropriate therapy.

The literature mentions a constellation of radiologic signs that should alert the treating surgeon preoperatively to suspect CNS lymphoma [8]. These signs are dependent on the patient's immune status, previous treatment administration, and whether the lymphoma arose primarily from the CNS or had disseminated there secondarily [8]. The classic appearance of PCNSL in an untreated immunocompetent patient is that of multiple supratentorial lesions that are close to the brain CSF interface; which may be either superficial (subpial) or deepseated (subependymal). They appear iso- to hypointense on T1-weighted images and hyperintense on T2-weighted images. Contrast uptake is usually avid and homogenous. The value of advanced MR imaging, consisting of spectroscopy and diffusion-weighted imaging, in the differentiation of these lesions from other tumors is limited owing to the similarity in profile with the highly cellular and actively proliferating glioblastomas and metastases [8, 9]. PCNSL shows restricted diffusion and MR spectroscopy shows elevated lipids with high choline/creatinine ratios [9]. These findings are consistent with other neoplastic lesions but may be of value in distinguishing it from infective or demyelinating processes [9]. 

Our case is unusual in that the lesion is isolated to the fourth ventricle, an extremely rare location that has only been documented in three other cases in literature [3–5] with an additional case having an associated lateral ventricle focus [10]. A solitary enhancing mass in an immunocompetent patient that forms a cast of the fourth ventricle should raise the suspicion of an ependymoma or, less likely, a subependymoma or medulloblastoma. The diagnosis of lymphoma in our case was indeed unforeseen and serves to reinforce the recommendation of previous authors to include it in the differential diagnosis of an intraventricular lesion in the appropriate setting, even it is if solitary [4, 5].

Survival outcomes mentioned for PCNSL have been uniformly disappointing despite recent advances and the often initial dramatic response to therapy [2, 11]. Current guidelines state that combination chemoradiation offers the best outcome, with median overall survival extended to 2–4 years and 5-year survival rate of 20–40% [2]. These compare favorably to those of radiation alone, which offered median survival of less than 18 months and a 5-year survival rate of less than 5%. Chemotherapy usually consists of high-dose methotrexate-based regimen while radiation consists of whole brain radiation therapy with or without local boost to the tumor bed [2].

The previously reported similar 3 cases are summarized in Table 1. The unusual location of the tumor in the surgically accessible posterior fossa, in addition to its isolation and subsequent hydrocephalus, may present a compelling indication for craniotomy and lymphoma resection even with a known diagnosis. This procedure is of limited penalty when compared to a supratentorial craniotomy for access of typical deep peri- or intraventricular lesions that account for the current consensus. Given the responsiveness of these tumors to chemoradiation, such surgery offers the possibility of rendering the patient shunt independent even with subtotal resection. The outcome is made more desirable with the recent report of systemic (cutaneous) PCNSL dissemination along the subcutaneous tract of a ventriculoperitoneal shunt and the difficulty in accessing the posterior fossa during an endoscopic third ventriculostomy [12].

## 4. Conclusion

We emphasize that PCNSL should be included in the differential diagnosis of intraventricular tumors, considering its implications on management planning. We also believe that resection is worthwhile, not contraindicated, in the context of a fourth ventricle lesion causing hydrocephalus. This will serve to spare the patient the morbidity of shunt dependency. 

## Figures and Tables

**Table 1:** Summary of the reported cases of 4th ventricular PCNSL in the literature.

Author (reference)	Age (yrs)/sex	Presentation	Radiology	Surgical treatment	CSF cytology	Adjuvant therapy	Survival
Werneck et al. [3]	17/F	Meningitis, diagnosis after mortem	Negative radiology but after mortem small tumor was found with carcinomatous meningitis	N/A	Positive	N/A	Diagnosis after mortem

Haegelen et al. [4]	33/F	Headache, vomiting with cerebellar signs	Tumor filling the 4th ventricle with no HCP	GTR	Negative	Cytarabine and steroids with stem cell transplantation with WBRT	7 months

Hill et al. [5]	69/F	Headache, vomiting	Homogenously enhancing tumor in the caudal fourth ventricle without hydrocephalus	Biopsy	N/A	IV and IT MTX	3 months

Current case	50/M	Headache, vomiting with drowsiness	Homogenously enhancing tumor in the caudal fourth ventricle with hydrocephalus	GTR	Negative	Regimen containing IV and IT MTX and radiotherapy	18 months

GTR: gross total resection; IV: intravenous; IT: intrathecal; MTX: methotrexate; N/A: not available.
